# Non-canonical role of “S6K1–SGK1” pathway in neuronal necroptosis following traumatic brain injury

**DOI:** 10.1016/j.gendis.2025.101876

**Published:** 2025-10-03

**Authors:** Shuchao Wang, Yating Tan, Minghai Hu, Meijuan Wang, Lu Liang, Xing Luo, Dan Chen, Bing Jiang, Ceshi Chen, Jufang Huang, Kun Xiong

**Affiliations:** a Department of Ophthalmology, The Second Xiangya Hospital of Central South University, Changsha, Hunan 410011, China; b Center for Medical Research, The Second Xiangya Hospital of Central South University, Changsha, Hunan 410011, China; c Department of Anatomy and Neurobiology, Xiangya School of Basic Medical Sciences, Central South University, Changsha, Hunan 410013, China; d Medical Imaging Center, Qingdao West Coast New District People's Hospital, Qingdao, Shandong 266100, China; e Hunan Key Laboratory of Ophthalmology, Changsha, Hunan 410011, China; f Hunan Clinical Research Center of Ophthalmic Disease, Changsha, Hunan 410011, China; g Department of Radiology, The Second Xiangya Hospital of Central South University, Changsha, Hunan 410011, China; h National Clinical Research Center for Mental Disorders, The Second Xiangya Hospital of Central South University, Changsha, Hunan 410011, China; i National Center for Mental Disorders, The Second Xiangya Hospital of Central South University, Changsha, Hunan 410011, China; j Kunming Institute of Zoology, Chinese Academy of Sciences, Kunming, Yunnan 650223, China; k Academy of Biomedical Engineering, Kunming Medical University, Kunming, Yunnan 650500, China; l The Third Affiliated Hospital, Kunming Medical University, Kunming, Yunnan 650118, China

**Keywords:** mTOR, Necroptosis, Oxidative stress, S6K1, SGK1, Traumatic brain injury

## Abstract

Traumatic brain injury (TBI) is characterized by high rates of death and disability. Necroptosis is reported to be involved in neuronal death after TBI. However, additional molecules and related mechanisms underlying necroptosis, particularly during TBI, remain to be elucidated. mTOR and two of its three substrates (4EBP1 and ULK1) are involved in necroptosis. However, direct evidence linking necroptosis to S6K, another key substrate of mTORC1, has been lacking. In this study, we aimed to investigate the regulated role of “S6K1-glucocorticoid-inducible kinase-1 (SGK1)” pathway in neuronal necroptosis after TBI. We first showed that the “S6K1–SGK1” pathway was activated during neuronal necroptosis in TNF-α/Smac mimics/Z-VAD-FMK-induced necroptotic cell model and mouse TBI model. Then, inhibition of the “S6K1–SGK1” pathway could decrease necroptosis by regulating the MLKL activation. Next, a rescue assay indicated that S6K1 may regulate necroptosis through modulating SGK1 expression, while not through binding with SGK1. Finally, S6K1 inhibition alleviated neuronal necroptosis, neuro-inflammation, and functional damage via SGK1 in mice after TBI. Our results showed a non-canonical role of “S6K1–SGK1” pathway in neuronal necroptosis following TBI in mice, which will provide a potential therapeutic target for necroptosis treatment in TBI and other necroptosis-related disorders.

## Introduction

Traumatic brain injury (TBI) is characterized by high rates of death and disability.^1^, ^2^, ^3^ The mortality rate for patients with severe TBI is approximately 40%, and the remaining patients also experience a significant decline in quality of life, which brings a heavy social and economic burden.^1^^,^^2^ Although several pathophysiological mechanisms of TBI, including inflammation, oxidative stress, and cell death, have been discovered, efficient therapeutic methods and molecular targets for TBI remain limited.^1^^,^^2^

Necroptosis, a type of programmed necrosis, is independent of caspases and is regulated by a complex comprising receptor-interacting protein-3 (RIP3), RIP1, and mixed lineage kinase domain-like protein (MLKL).^4^, ^5^, ^6^, ^7^ After MLKL dissociates from the necrosome, it translocates to the plasma membrane, inducing the formation of membrane pores and leading to necroptosis.^4^, ^5^, ^6^, ^7^ The classical necroptosis can be induced by the combined application of tumor necrosis factor alpha (TNF-α), second mitochondrial activator of caspases (Smac) mimics, and caspase inhibitor Z-VAD-FMK (TSZ) through Fas-associated via death domain (FADD).^4^^,^^5^^,^^8^ In addition, necroptosis is stimulated under various conditions such as trauma, oxidative stress, and ischemic injury.^4^^,^^5^^,^^9^^,^^10^ Although the “RIP3–MLKL” pathway plays a key role in necroptosis, the upstream and downstream pathways involved in necroptosis pathogenesis remain unclear and require further study. Numerous studies have demonstrated a strong connection between necroptosis and TBI, and some have explored the molecular mechanisms involved.^11^, ^12^, ^13^ Smad ubiquitin regulatory factor 1 (Smurf1), stimulator of interferon gene (STING), transcription factor EB (TFEB), and a series of microRNAs modulate neuronal necroptosis following TBI.^6^^,^^14^^,^^15^ The application of necrostatin-1 (Nec-1), a broad-spectrum necroptotic inhibitor, can reduce neuronal death following TBI, suggesting that it may be a promising therapeutic strategy for TBI.^16^^,^^17^ However, additional molecules and related mechanisms underlying necroptosis, particularly during TBI, remain to be elucidated.

The mammalian target of rapamycin (mTOR) is a serine/threonine protein kinase that regulates fundamental cellular processes, including cell proliferation, growth, and development, by integrating various signals, such as glucose, amino acids, and cytokines.^18^^,^^19^ mTOR forms two distinct complexes with different proteins: mTOR complex 1 (mTORC1), which includes mLST8, PRAS40, Raptor, and DEPTOR; and mTORC2, which includes mLST8, mSin1, Protor, Rictor, and DEPTOR.^18^^,^^19^ Recent studies suggest that mTORC1 is involved in the regulation of necroptosis.^7^^,^^20^^,^^21^ Excess amino acids or inhibition of tuberous sclerosis 1 (TSC1) can activate RIP3 and MLKL through mTORC1, ultimately triggering necroptosis and disrupting homeostasis in intestinal epithelial cells.^21^

mTORC1 plays a classical role in protein synthesis and autophagy by phosphorylating its three major substrates: eukaryotic translation initiation Factor 4E (eIF4E) binding protein-1 (4EBP1), p70 ribosomal S6 kinase (S6K), and UNC-51-like kinase 1 (ULK1).^18^^,^^19^ Recent evidence suggests that ULK1 overexpression promotes autophagy and inhibits necroptosis by enhancing receptor-interacting protein kinase 1 (RIPK1) phosphorylation at Ser357.^22^^,^^23^ In addition, eIF4E has been shown to mediate necroptosis and plays a critical role in triple-negative breast cancer.^24^^,^^25^ Consistent with these findings, our recent results showed that “4EBP1–eIF4E” axis is involved in regulating necroptosis in ischemic brain injury.^26^ Specifically, 4EBP1 overexpression and eIF4E inhibition could suppress necroptosis by down-regulating the RIP3–MLKL pathway. Furthermore, this pathway appears to operate through a positive feedback circuit, in which the RIPK3–MLKL signaling promotes degradation of 4EBP1 via the ubiquitin system, thereby enhancing eIF4E activity and amplifying RIP3 and MLKL activation. Finally, excessive eIF4E activation, likely driven by this feedback circuit, induces chemokine and cytokine production, potentially contributing to necroptosis-associated inflammation. However, direct evidence linking necroptosis to S6K, another key substrate of mTORC1, has been lacking. In our recent study, we observed that S6K1 expression significantly changed during the necroptotic process.^26^ Notably, our previous study showed that p90 ribosomal S6 kinase 3 (RSK3) regulated necroptosis in retinal neurons.^27^ Besides, other studies have shown that RSK1/2 also play a crucial role in necroptosis in TNF-treated L929 cells and in cecum damage, through activation of pyruvate dehydrogenase kinase-1 (PDK1).^28^^,^^29^ Given that both RSK and S6K belong to the ribosomal protein kinase A, G, and C (AGC) family and have similar biological functions,^30^^,^^31^ we speculate that S6K1 may also play a regulatory role in necroptosis.

In addition, we screened the underlying regulated molecules in necroptosis related to the mTOR pathway and found that serum levels of glucocorticoid-inducible kinase-1 (SGK1) increased during necroptosis. SGK1 belongs to the AGC family and is activated by mTORC2, which is stimulated by growth factors and cellular and oxidative stress.^32^^,^^33^ Notably, several studies have shown that RSK, S6K1, and SGK1 are activated by PDK1 in response to insulin and growth factor (IGF) signaling, and they regulate a variety of cellular processes.^32^^,^^33^ However, further experiments have shown that both wild-type and phosphorylated forms of S6K1 can bind to PDK1. In contrast, PDK1 binds to SGK1 only when serine 422 is phosphorylated, and not to the wild-type SGK1.^33^, ^34^, ^35^ While the roles of RSK and PDK1 in necroptosis have been previously reported, however, it remains unclear whether S6K1 and SGK1 also participate in necroptotic regulation or interact with each other in this context. Further studies are needed to clarify these potential regulatory mechanisms.

In this study, we observed a novel and non-canonical role of the S6K1–SGK1 pathway in necroptosis in TBI, unlike the classical role of S6K1 and SGK1 in diverse cellular processes, such as cell proliferation and apoptosis. These findings provide a potential therapeutic target for the treatment of TBI and other necroptosis-related injuries.

## Materials and methods

### HT22 neuronal cell line and necroptotic model

HT22 cells were obtained from the American Type Culture Collection (Manassas, USA) and cultured in Dulbecco's Modified Eagle Medium (Gibco, New York, USA) with fetal bovine serum (Gibco) under 37 °C and 5% CO_2_. A commercial TSZ kit (Beyotime, Beijing, China) was added for different time points to induce necroptosis. Nec-1 (20 μM), PF4708671 (20 μM), and GSK650394 (15 μM) were all from MedChemExpress (New Jersey, USA) and were added 30 min before TSZ injury.

### Mouse TBI model, drug application, and tissue preparation

Animal assays were approved by the animal research committee of the Second Xiangya Hospital of Central South University (approval number: 2021136). Eight-week-old male C57/BL6 mice were randomly used for the TBI model as previously described.^36^ Briefly, TBI injury was established by a method of controlled cortical impact. After anesthetization, the mouse skull was exposed and underwent a 5 mm craniotomy, positioned 2 mm to the right of the sagittal suture and 2 mm posterior to the coronal suture. A 4 mm cranial window was drilled at the marked site, carefully removing bone fragments without damaging brain tissue. The right hemisphere was selected for injury. Under a stereotaxic apparatus (Rodent Stereotaxic System, RWD, China), the impact coordinates were identified relative to a depth of 2 mm, delivered by a 2 mm impacting tip (Small Animal Craniocerebral Precision Impactor, RWD, China) at a velocity of 3.5 m/s with a dwell time of 0.2 s. Sham mice only underwent scalp incision. Hemostasis was achieved using sterile cotton swabs. The bone window was sealed, and the scalp was sutured and disinfected with iodophor. Mice were placed on a 37 °C heating pad until fully awake. The TBI severity was classified as moderate, assessed using the modified Neurological Severity Score (mNSS) to evaluate neurological deficits.

For the inhibitor treatment group, mice were intraperitoneally injected with the S6K1 inhibitor PF4708671 (12.5 mg/kg) every 12 h after TBI injury for 3 days^37^ (Fig. 5H). Adeno-associated virus (AAV) shRNA targeting S6K1 (targeting sequence: GGAAGAUAUUUGCCAUGAATT) was constructed and packaged by OBio (Shanghai, China). Two μL 10^12^ VG/mL AAV–shS6K1 was injected into the right lateral ventricles (depth: 3.0 mm) at a speed of 1 μL/min. The needle was kept in place for 2 min after the injection was complete. Follow-up experiments were performed 2 weeks later.

**Figure 1:**
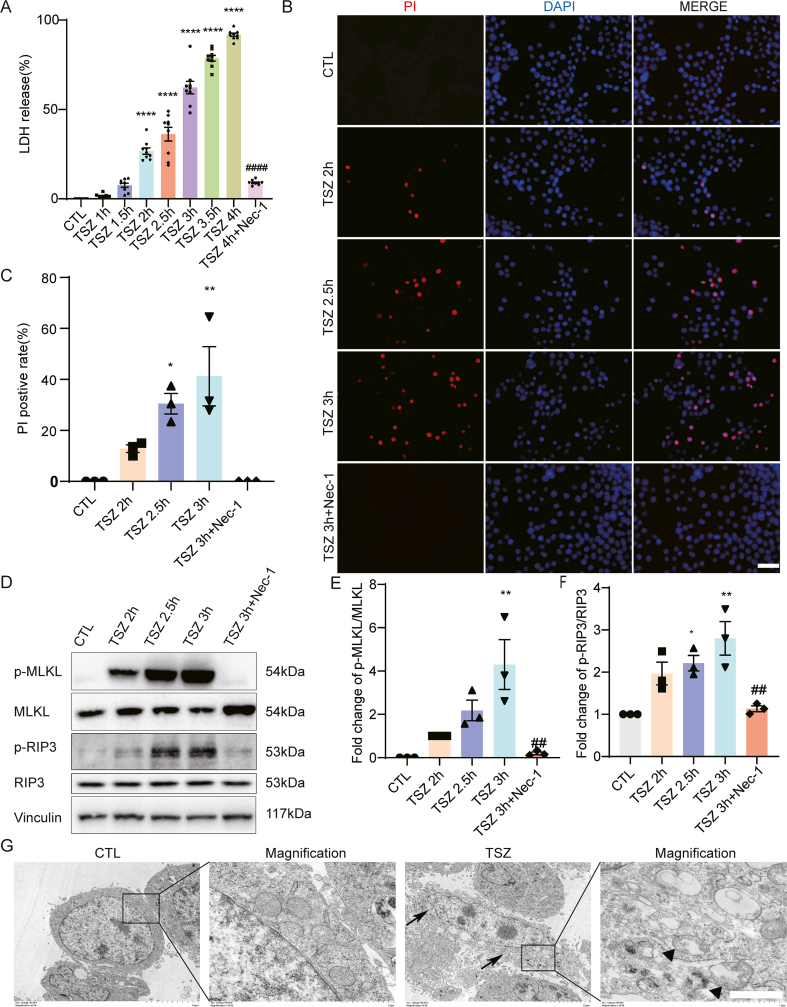
TSZ induces necroptosis in HT22 cells. **(A)** LDH assay indicated an increased percentage of cell death following TSZ application, which was reduced by Nec-1 pretreatment before TSZ application. **(B)** PI staining (red) indicated the increased changes of necrotic cells after TSZ application and the decreased changes of necrotic cells after pretreatment with Nec-1 before TSZ application. Nuclei were stained with DAPI (blue). Scale bar: 50 μm. **(C)** Statistical results of the PI-positive necrotic cells. **(D)** Western blotting showed the increased changes of p-MLKL/MLKL and p-RIP3/RIP3 expression in HT22 cells after TSZ application or pretreatment with Nec-1 before TSZ application. **(E, F)** Statistical results of Western blotting. **(G)** Representative transmission electron microscopic images showed necroptosis after TSZ treatment. The black arrowhead indicates plasma membrane rupture. The triangle indicates mitochondrial swelling in TSZ-treated HT22 cells. Scale bar: 10 μm. *n* = 3 or 9. ∗*P* < 0.05, *∗∗P* < 0.01, and *∗∗∗∗P* < 0.0001 versus the CTL group. ^*##*^*P* < 0.01 and ^*####*^*P* < 0.0001 versus the TSZ 3 h group. Two-tailed unpaired Student's *t*-test was used for Nec-1 comparison.

**Figure 2:**
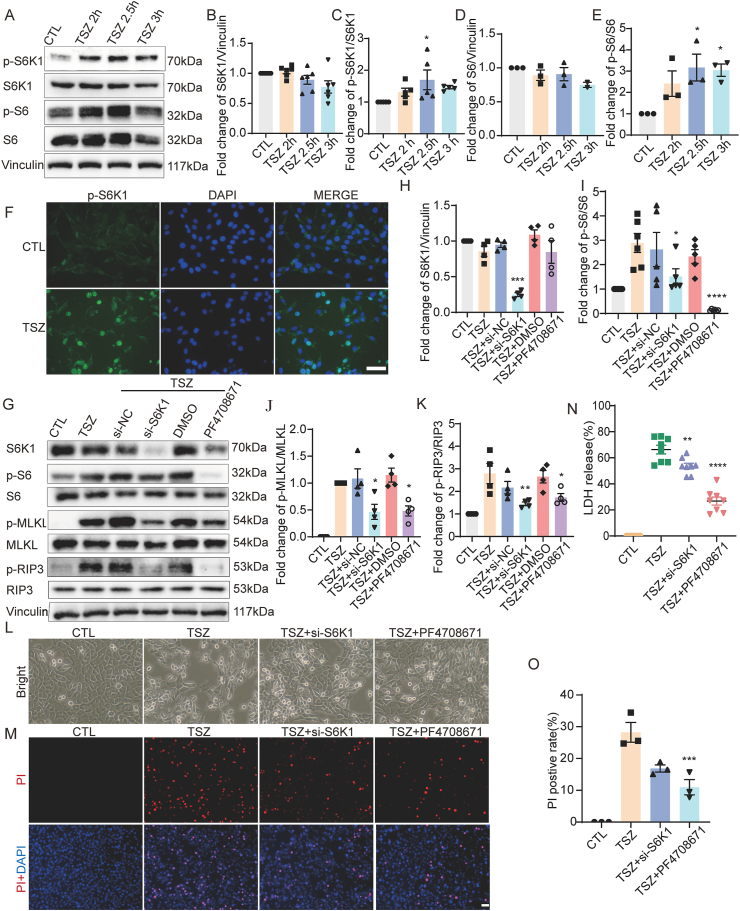
S6K1 knockdown and inhibition reduce necroptosis in HT22 cells. **(A, G)** Western blotting showed increased levels of p-S6K1/S6K1, p-S6/S6, p-MLKL/MLKL, and p-RIP3/RIP3 expression following TSZ application. These increases were attenuated by pretreatment with *S6K1* siRNA and an S6K1 inhibitor before TSZ application. **(B–E, H–K)** Statistical results of Western blotting. **(F)** Enhanced immunofluorescence intensity of p-S6K1 in HT22 cells after TSZ application. **(L)** Phase-contrast images showed changes in necrotic cells following TSZ application, as well as after pretreatment with *S6K1* siRNA and inhibitor before TSZ application. Microscopic magnification, 20 ×. **(M)** PI staining (red) indicated the decreased changes in necrotic cells after pretreatment with *S6K1* siRNA and inhibitor before TSZ application compared with TSZ application. Nuclei were stained with DAPI (blue). **(N)** LDH assay indicated the decreased percentage of cell death after pretreatment with *S6K1* siRNA and inhibitor before TSZ application compared with TSZ application. **(O)** Statistical results of the PI-positive necrotic cells. *n* = 3, 4, 5, 6, or 8. ∗*P* < 0.05, *∗∗P* < 0.01, *∗∗∗P* < 0.001, and *∗∗∗∗P* < 0.0001 versus the CTL or TSZ group. Scale bar: 50 μm.

**Figure 3:**
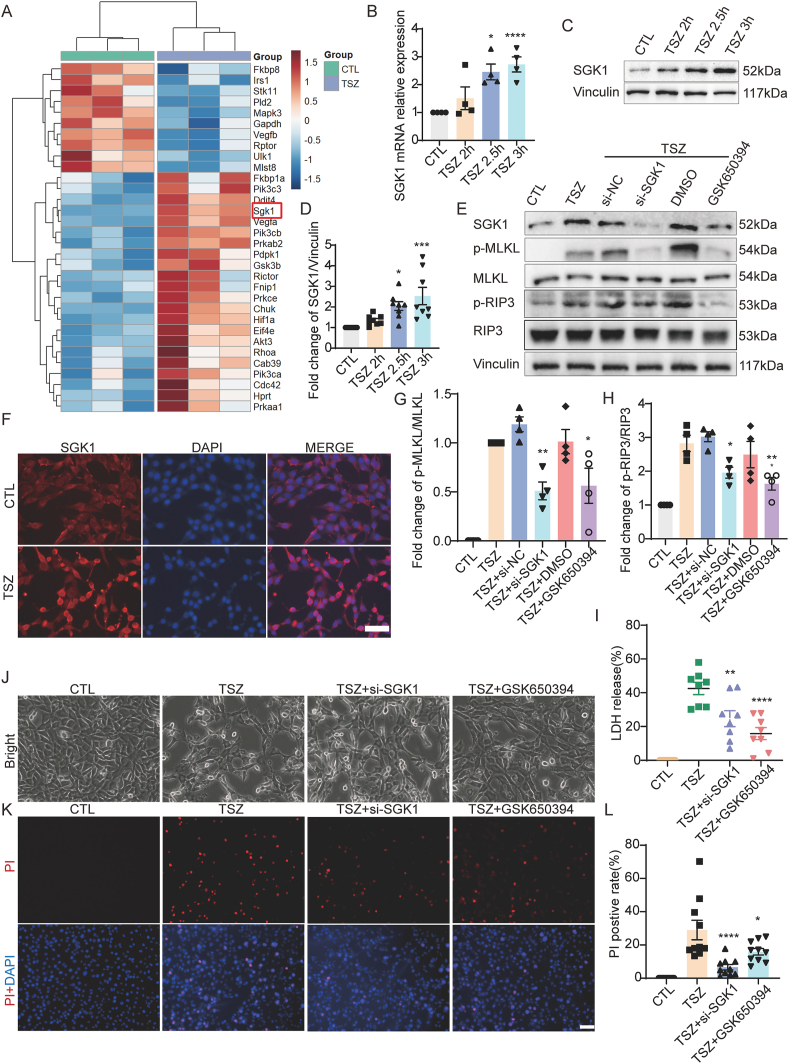
SGK1 knockdown and inhibition reduce necroptosis in HT22 cells. **(A)** The heatmap focused on the mTOR pathway in HT22 cells after TSZ application. **(B)** Increases relative changes of *SGK1* mRNA after TSZ treatment. **(C, E)** Western blotting showed increased SGK1 levels following TSZ treatment, while the expression of SGK1, p-MLKL/MLKL, and p-RIP3/RIP3 decreased in HT22 cells after pretreatment with *SGK1* siRNA and inhibitor before TSZ application. **(D, G, H)** Statistical results of Western blotting. **(F)** Enhanced immunofluorescence intensity of SGK1 in HT22 cells after TSZ application. **(I)** LDH assay indicated the decreased percentage of cell death after pretreatment with *SGK1* siRNA and inhibitor before TSZ application compared with TSZ application. **(J)** Phase-contrast images showed the changes in necrotic cells after TSZ application or pretreatment with *SGK1* siRNA and inhibitor before TSZ application. Microscopic magnification, 20 ×. **(K)** PI staining (red) indicated the decreased changes in necrotic cells after pretreatment with *SGK1* siRNA and inhibitor before TSZ application compared with TSZ application. Nuclei were stained with DAPI (blue). **(L)** Statistical results of the PI-positive necrotic cells. *n* = 4, 8, or 10. ∗*P* < 0.05, *∗∗P* < 0.01, *∗∗∗P* < 0.001, and *∗∗∗∗P* < 0.0001 versus the CTL or TSZ group. Scale bar: 50 μm.

**Figure 4:**
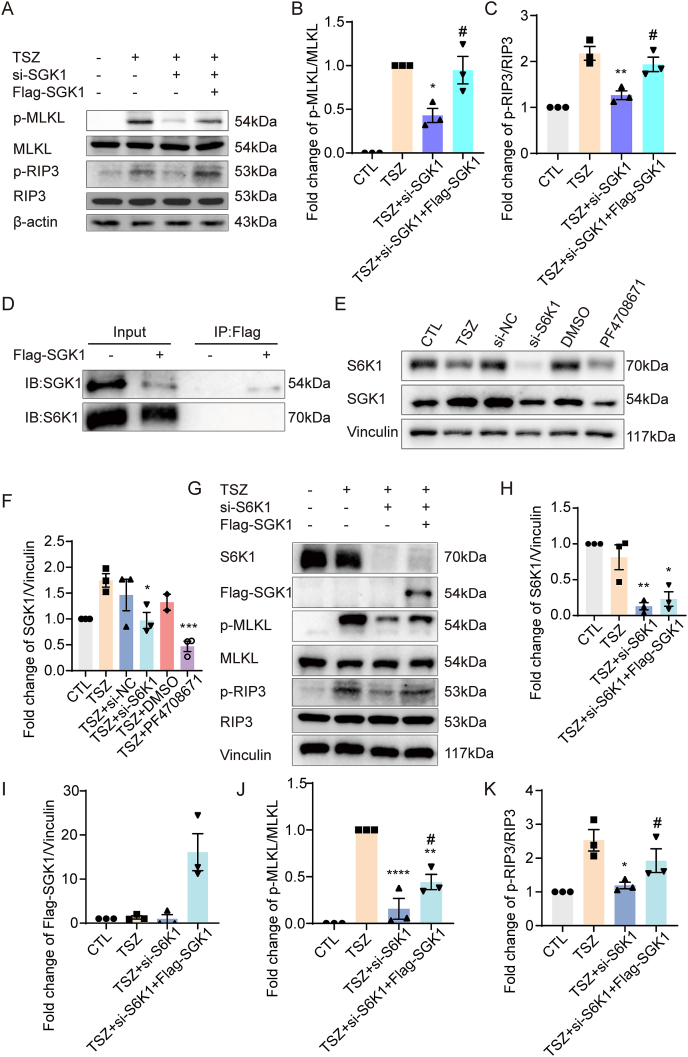
S6K1 regulates necroptosis through SGK1 in HT22 cells. **(A, E, G)** Western blotting showed increased changes in pMLKL/MLKL and pRIP3/RIP3 after pretreatment with SGK1 overexpression before TSZ application compared with pretreatment with *SGK1* siRNA, decreased changes in S6K1 and SGK1 expression after *S6K1* siRNA and inhibitor, decreased changes in S6K1, SGK1, p-MLKL/MLKL, and p-RIP3/RIP3 after pretreatment with *S6K1* siRNA before TSZ application compared with TSZ application, and increased changes in S6K1, SGK1, p-MLKL/MLKL, and p-RIP3/RIP3 after pretreatment with SGK1 overexpression before TSZ application compared with pretreatment with *S6K1* siRNA. **(B, C, F, H–K)** Statistical results of Western blotting. **(D)** Co-immunoprecipitation results showed no interaction between S6K1 and SGK1. A two-tailed unpaired Student's *t*-test was used to compare “TSZ + *SGK1* siRNA” or “TSZ + *S6K1* siRNA”. *n* = 3. ∗*P* < 0.05, *∗∗P* < 0.01, *∗∗∗P* < 0.001, and *∗∗∗∗P* < 0.0001 versus the TSZ group. ^#^*P* < 0.05 versus the TSZ + *S6K1* siRNA group. Two-tailed unpaired Student's *t*-test was used to compare the “TSZ + *S6K1* siRNA” group and “TSZ + *S6K1* siRNA + Flag-SGK1” group.

**Figure 5:**
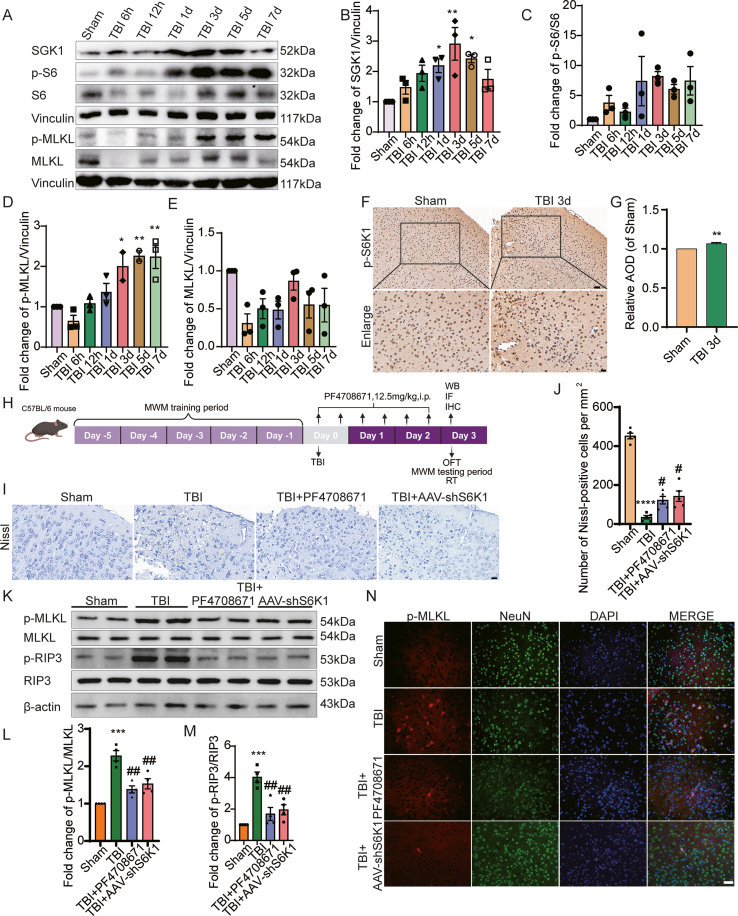
S6K1 inhibition alleviates neuronal necroptosis through SGK1 following TBI in mice. **(A, K)** Western blotting showed increased expression of p-S6/S6, SGK1, p-MLKL/MLKL, and p-RIP3/RIP3 in the cortex following TBI. These changes were attenuated by S6K1 inhibitor treatment and *S6K1* knockdown, as shown by decreased p-MLKL/MLKL and p-RIP3/RIP3 levels compared with the TBI group. **(B–E, L, M)** Statistical results of Western blotting. **(F)** Immunohistochemistry staining of p-S6K1 in the right cortex of mouse brain after TBI. Scale bar: 20 μm. **(G)** Statistical results of immunohistochemistry staining for the increased S6K1 expression. **(H)** Schematic illustration of protein detection and behavior paradigm following p-S6K1 inhibitor injection. **(I)** Nissl staining of decreased neurons after TBI, and increased neurons after treatment with S6K1 inhibitor and knockdown compared with TBI. Scale bar: 20 μm. **(J)** Statistical results of Nissl staining for the numbers of neurons. **(N)** Enhanced immunofluorescence intensity of p-MLKL in the right cortex of mouse brain after TBI, and decreased immunofluorescence intensity after treatment with S6K1 inhibitor and knockdown compared with TBI. Scale bar: 50 μm. Two-tailed unpaired Student's *t*-test was used for (G). *n* = 3 or 4. ∗*P* < 0.05, *∗∗P* < 0.01, *∗∗∗P* < 0.001, and *∗∗∗∗P* < 0.0001 versus the Sham group. ^*#*^*P* < 0.05 and ^*##*^*P* < 0.01 versus the TBI group.

For immunohistochemistry staining, 3 days after TBI, 4 or 5 mouse brains from each group were removed and fixed in paraformaldehyde (Beyotime, Beijing, China), which were then equilibrated in sucrose solutions (Beyotime), embedded in paraffin, and subjected to sectioning. For Western blotting, 3 days after TBI, the skulls from 3 or 4 mouse brains in each group were carefully removed to expose the brain. The brain was washed twice with pre-cold phosphate-buffered saline. The right cerebral cortical tissue was dissected and immediately transferred into a sterile EP tube. Proteins from mouse brains were extracted by the ice-cold lysis buffer (Beyotime) with protease and phosphatase inhibitors (Beyotime).

### Propidium iodide (PI) staining

PI (Thermo Fisher, Waltham, USA) staining was used to evaluate the percentage of necrotic cells. HT22 cells were washed 3 times with phosphate-buffered saline at the observed time points and then stained with PI for 20 min. Then, the cells were washed again and covered by a Mounting Medium with 4′,6-diamidino-2-phenylindole (DAPI) (MMD) (Thermo Fisher) and captured by a fluorescence microscope (Zeiss, Jena, Germany). The PI-positive cells were evaluated by Image J (National Institutes of Health, Baltimore, USA). PI-positive cells were identified in a double-blind manner, and the number of positive cells was normalized to the total number of imaged cells.

### Lactate dehydrogenase (LDH) release assay

The LDH (Beyotime) assay was used to evaluate the ratio of cell death. Cell supernatants were first centrifuged, collected, and incubated with pre-treated reagent mixture, followed by detection of the absorbance at the wavelength of 490 nm. The ratio of cell death was the ratio of the absorbance of (intervenient cells–control cells) to the absorbance of (massive LDH value–control cells).

### Transmission electron microscopy

Transmission electron microscopy was used to evaluate the necroptotic morphological changes. Cells were fixed with 2% osmium tetroxide, and then dehydrated and embedded with pure epoxy resin. The necroptotic morphological changes were defined with a transmission electron microscope (HT7800 from HITACHI, Tokyo, Japan).

### Western blotting and co-immunoprecipitation

Total proteins from HT22 cells or mouse brains were extracted by the lysis buffer (Beyotime) with protease and phosphatase inhibitors (Beyotime), separated by the 10% SDS-PAGE gel, and then transferred to the 0.22 μm PVDF membrane (Merk Millipore, Darmstadt, Germany). The membrane with protein was blocked by the blocking buffer (Beyotime), incubated with primary antibodies: MLKL (1:5000, Abcam, Cambridge, UK), p-MLKL (1:5000, Abcam), S6K1 (1:1000, Beyotime), p-S6K1 (1:1000, Beyotime), S6 (1:5000, Proteintech, Wuhan, China), p-S6 (1:5000, Proteintech), SGK1 (1:5000, Proteintech), Flag (1:5000, Abcam) and Vinculin (1:200, Santa Cruz, Dallas, USA) at 4 °C overnight, and incubated with horseradish peroxidase-labeled secondary antibodies (1:5000, Beyotime) for 2 h. Finally, the blots were exposed and captured by a chemiluminescence instrument (Bio-Rad, Hercules, USA). For the co-immunoprecipitation assay, total proteins from HT22 cells were extracted by a non-denaturing lysis buffer (Beyotime), and then incubated with Flag antibody (1:100) coupled with magnetic beads. Last, the complex-conjugated proteins were analyzed by Western blotting.

### Immunofluorescence and immunohistochemistry staining

Paraformaldehyde-fixed HT22 cells or dewaxed brain sections were blocked by horse serum, incubated with primary antibodies: p-S6K1 (1:100), SGK1 (1:200), p-MLKL (1:200), NeuN (1:200), Iba1 (1:200), and GFAP (1:200), and Alexa-conjugated secondary antibody (1:500, Jackson ImmunoResearch, West Grove, USA) or horseradish peroxidase-conjugated secondary antibody (1:200, Beyotime). Finally, cells or sections were covered by MMD and captured by a fluorescence microscope at the same settings. Image acquisition and processing were performed with Zen Blue software (Zeiss). The exposure time was kept constant for each protein. Five random areas in three random sections were captured by a Zeiss AXIO Scope A1. Quantitative assessments were performed using ImageJ software to calculate the integrated density of p-S6K1, GFAP, and Iba1 *in vivo*.

### RNA sequencing and quantitative real-time PCR

Total RNAs from HT22 cells were extracted by Trizol (Invitrogen, California, USA). RNA sequencing was accomplished by the BGI Genomics (Shenzhen, China). The differentially expressed genes were plotted with a heatmap in R software. For quantitative PCR assay, the 2^−ΔΔCt^ method was used. The SYBR Green PCR kit (Transgene, Beijing, China) was used for the quantitative PCR procedure. The gene expression was normalized to β-actin. The mRNA primers are as follows: *Sgk1* forward primer, 5′-CAAATCAACCTGGGTCCGTC-3'; *Sgk1* reverse primer, 5′-AAGAACCTTTCCAAAACTGCCC-3'; *β-actin* forward primer, 5′-TCCTATGTGGGTGACGAGGC-3'; *β-actin* reverse primer, 5′-TACATGGCTGGGGTGTTGAA-3'.

### Transfection approach and rescue assay

*S6K1* siRNA (targeting sequence: GCACCUGCGGAUGAAUCUATT) and *SGK1* siRNA (targeting sequence: GTCTGTACATTGGGTTATA) were provided by Genechem Biotechnology (Shanghai, China). The full length of SGK1 was cloned into the pcDNA3.1-Flag vector constructed by Fenghui Biotechnology (Changsha, China). For transfection procedures, 3 μL siRNAs/5 μg plasmid and 7.5 μL Lipofectamine RNAiMax/10 μL Lipofectamine 3000 (Thermo Fisher) were incubated in the opti-MEM (Gibco), respectively, and then subjected to mixing. Last, adding the mixtures to the cultures for transfection. For the upstream/downstream regulated relationship or the rescue assay, HT22 cells were transduced with *S6K1* or *SGK1* siRNA for 4 h, and then transduced with SGK1 plasmid. Last, the cells were injured with TSZ.

### Nissl staining

The brain sections were dewaxed and stained with Nissl staining solution (Beyotime) for 10 min. The stained brain sections were mounted on slides, covered by MMD, and examined under a microscope (Zeiss).

### Rotarod test

The rotarod treadmill (Med Associates, Albans, USA) was used to assess the motor coordination of the animals. Mice were placed on the rotarod and pre-trained for 5 min at an increased speed of 5–40 rpm. Last, the time that mice remained on the rotating bar was recorded.

### Open-field test

Mice were placed in the central area of an open arena (40 cm in length, 40 cm in width, and 30 cm in height), allowing them to freely explore the environment for 5 min. The movements were monitored by an overhead camera. The total travel distance and the distance traveled within the central square and perimeter were analyzed utilizing a Tracking Software (Noldus Information Technology, Wageningen, Netherlands).

### Morris water maze tests

The Morris water maze apparatus comprised a circular pool with a 1-m diameter, featuring a submerged platform hidden in one of its quadrants. The entire experiment was divided into two parts: the positioning navigation experiment and the spatial exploration experiment. During the first five days, the platform was positioned in the southwest quadrant, submerged 1 cm below the water surface. Mice were sequentially placed along the pool wall at four different starting points (one per trial) and trained four times daily. In each trial, mice were allowed 90 s to locate the platform. If unsuccessful within this timeframe, they were gently guided to the platform and permitted to remain on it for 15 s for spatial memory consolidation. On the sixth day, the platform was removed. Mice were introduced to the pool along the northeast quadrant wall and allowed to freely explore the pool for 90 s. The escape latency, the number of platform crossings, and exploration times in the target quadrant for mice were recorded with an image acquisition and analysis system.

### Modified neurological severity scoring

Neurological deficits were assessed by mNSS. The score was performed by blinded independent persons to evaluate the sensory, motor, reflex, and balance functions. The score was divided into 0–18, with normal mice scored as 0 and maximal neurological deficit scored as 18. Cumulative scores of 1–6, 6–12, and 13–18 indicate mild, moderate, and severe deficit, respectively.

### Analysis of brain edema

Brain water content was measured in 3-mm coronal sections of the ipsilateral cortex, centered upon the impact site. Sections were immediately weighed as wet weight, and then dehydrated at 100 °C for 24 h to weigh the dry weight. The water content was calculated as percent (%) = [(wet weight–dry weight)/wet weight] × 100.

### Enzyme-linked immunosorbent assay (ELISA)

Cerebral cortex tissues were lysed on ice and centrifuged to obtain the supernatant. Tissue Tnf-α and interleukin-1beta (Il-1β) levels were analyzed by ELISA kit according to the manufacturer's instructions (Beyotime).

### Statistical analysis

The results were analyzed by GraphPad Prism 8 (GraphPad Software, San Diego, USA). The data were shown as mean ± standard error of the mean. Comparisons between two independent groups were performed using a two-tailed, unpaired *t*-test. Two-way analysis of variance (ANOVA) with Tukey's multiple comparisons test was used to analyze differences among three or more groups when the data were normally distributed, and nonparametric Mann–Whitney *U* tests were used for groups if the data were not normally distributed. *P* < 0.05 was considered statistically significant.

## Results

### TSZ induces necroptosis in HT22 cells

We investigated whether TSZ induced necroptosis in HT22 cells. The LDH assay results showed that the number of dead HT22 cells gradually increased at all observed time points and could be inhibited by Nec-1, a specific inhibitor of necroptosis (Fig. 1A). In subsequent experiments, we simplified the observation time points and selected 3 h as the main intervention time point. The PI staining results further showed that necrotic HT22 cells increased after TSZ treatment and were inhibited by Nec-1 (Fig. 1B and C). Finally, Western blotting showed that the ratios of p-MLKL/MLKL and p-RIP3/RIP3, which are markers of necroptotic activation, significantly increased after TSZ treatment and were inhibited by Nec-1 (Fig. 1D–F). To further observe the ultrastructure of cells, transmission electron microscopy was used, which revealed necroptotic characteristics such as mitochondrial swelling, plasma membrane rupture, and progressively translucent cytoplasm in TSZ-treated cells (Fig. 1G). These results indicated that TSZ specifically induced necroptosis in HT22 cells.

### S6K1 knockdown and inhibition reduce necroptosis in HT22 cells

In this study, we investigated the regulatory role of S6K1 in necroptosis. Western blotting results showed that S6K1 expression was not significantly altered, while the ratio of p-S6K1/S6K1 was significantly increased in necroptotic HT22 cells (Fig. 2A–C), suggesting that S6K1 is activated in the necroptotic process. Furthermore, the ratio of p-S6/S6, a marker of S6K1 activation, significantly increased in necroptotic HT22 cells (Fig. 2A, D, E). These results were confirmed by immunofluorescence staining, which showed enhanced p-S6K1 fluorescence intensity, especially in the nuclei of necroptotic HT22 cells (Fig. 2F). To determine the regulated role of S6K1 in necroptosis, *S6K1* siRNA was used to silence S6K1 expression and to determine its role in necroptosis (Fig. 2G and H). In addition, the S6K1 inhibitor PF4708671 was used to inhibit S6K1 activity, as indicated by the decreased p-S6/S6 ratio (Fig. 2G, I). As expected, S6K1 silencing and inhibition significantly inhibited MLKL and RIP3 activation (Fig. 2G, J, K). In addition, phase-contrast images showed that TSZ triggered an increase in necrotic cells, characterized by cell swelling and rupture, which could be inhibited by S6K1 silencing and inhibition (Fig. 2L). Finally, PI and LDH results showed that necroptotic HT22 cells decreased after S6K1 silencing and inhibition (Fig. 2M−O). Collectively, these results indicate that S6K1 is an important regulator of necroptosis.

### SGK1 knockdown and inhibition reduce necroptosis in HT22 cells

RNA sequencing was first performed to determine the transcriptional changes in potential upstream and downstream molecules in the necroptotic process, especially in the mTOR-related necroptotic pathway. As mentioned in the introduction, SGK1, S6K1, and RSK were activated and combined with PDK1, and SGK1 also increased during the necroptotic process, as indicated by the RNA sequencing results (Fig. 3A). We further used quantitative PCR and Western blotting to verify the RNA sequencing results, which showed that SGK1 expression was increased at both the transcriptional and translational levels (Fig. 3B–D). Immunofluorescence staining results showed enhanced SGK1 fluorescence intensity, especially in the cytoplasm of necroptotic HT22 cells (Fig. 3F). To determine the regulatory role of SGK1 in necroptosis, *SGK1* siRNA and the inhibitor GSK650394 were used to silence SGK1 expression and inhibit its SGK1 activity (Fig. 3E). As expected, SGK1 silencing and inhibition significantly inhibited MLKL and RIP3 activation (Fig. 3E, G, H). In addition, phase-contrast images showed that SGK1 silencing and inhibition suppressed necrotic cell growth (Fig. 3J). PI and LDH results also showed that the number of necroptotic HT22 cells decreased after SGK1 silencing and inhibition (Fig. 3I, K, L). Finally, we performed a rescue assay to further overexpress SGK1 after SGK1 knockdown, to validate the effects of SGK1 up-regulation on necroptosis. Rescue assays showed that SGK1 overexpression restored the activation of pMLKL/MLKL and pRIP3/RIP3, which was inhibited by SGK1 knockdown (Fig. 4A–C). Overall, these results indicate that SGK1 is an important regulator of necroptosis.

### S6K1 regulates necroptosis through SGK1 in HT22 cells

To further investigate the relationship between S6K1 and SGK1, and whether S6K1 is an up-regulator of SGK1 in the necroptotic process, we performed co-immunoprecipitation and rescue assays. As mentioned in the *Introduction*, SGK1, S6K1, and RSK are activated and combine with PDK1. However, our co-immunoprecipitation results showed no direct or indirect binding between S6K1 and SGK1 (Fig. 4D). *S6K1* siRNA and the inhibitor PF4708671 were used to silence S6K1 expression and inhibit the S6K1 activity (Fig. 4E and F). S6K1 silencing and inhibition significantly decreased SGK1 expression (Fig. 4E and F), indicating that S6K1 may up-regulate SGK1 expression in HT22 cells. Finally, HT22 cells were transduced with *S6K1* siRNA, co-transduced with SGK1 over-expressed plasmid, and exposed to TSZ (Fig. 4G–K). *S6K1* siRNA inhibited TSZ-induced MLKL and RIP3 activation, which was enhanced by SGK1 overexpression (Fig. 4G, J, K), indicating that S6K1 may be an up-regulator of SGK1 in necroptotic HT22 cells. These results indicate that S6K1 may regulate necroptosis by modulating SGK1 expression, but not by binding to SGK1.

### S6K1 inhibition alleviates neuronal necroptosis through SGK1 following TBI in mice

Next, we used the TBI mouse model to verify the regulatory role of S6K1 in neuronal necroptosis *in vivo*. We found that the SGK1 protein, the ratio of p-S6/S6, a marker of S6K1 activation, and p-MLKL/MLKL, a marker of necroptotic activation, were significantly increased in TBI mice (Fig. 5A–E). S6K1 activation peaked at 1 day after TBI, and SGK1 protein levels increased and MLKL activation peaked at 3 days after TBI (Fig. 5B–D). The immunohistochemistry staining results also indicated elevated p-S6K1 expression at 3 days after TBI (Fig. 5F and G). Based on these findings, we selected 3 days post-TBI as the primary time point for subsequent intervention assays. Nissl staining results showed that neurons in the regions surrounding the TBI showed extensive damage, nuclear solidification, cytoplasmic contraction, and decreased numbers (Fig. 5I and J). After the application of the S6K1 inhibitor PF4708671 and injection of AAV-shS6K1 to knock down *S6K1*, neuronal damage was partially inhibited, and the numbers were partially restored (Fig. 5I and J). Furthermore, the ratios of p-MLKL/MLKL and p-RIP3/RIP3 also decreased after application of the S6K1 inhibitor and AAV-shS6K1 compared with those in the TBI group (Fig. 5K–M). Additionally, the immunofluorescence staining results demonstrated that the TBI group had more p-MLKL/NeuN-positive cells, which were significantly reduced after the application of the S6K1 inhibitor and AAV-shS6K1 (Fig. 5N). Taken together, these results indicated that S6K1 inhibition alleviated neuronal necroptosis following TBI in mice.

### S6K1 inhibition alleviates neuro-inflammation and functional damage after TBI in mice

**Figure 6:**
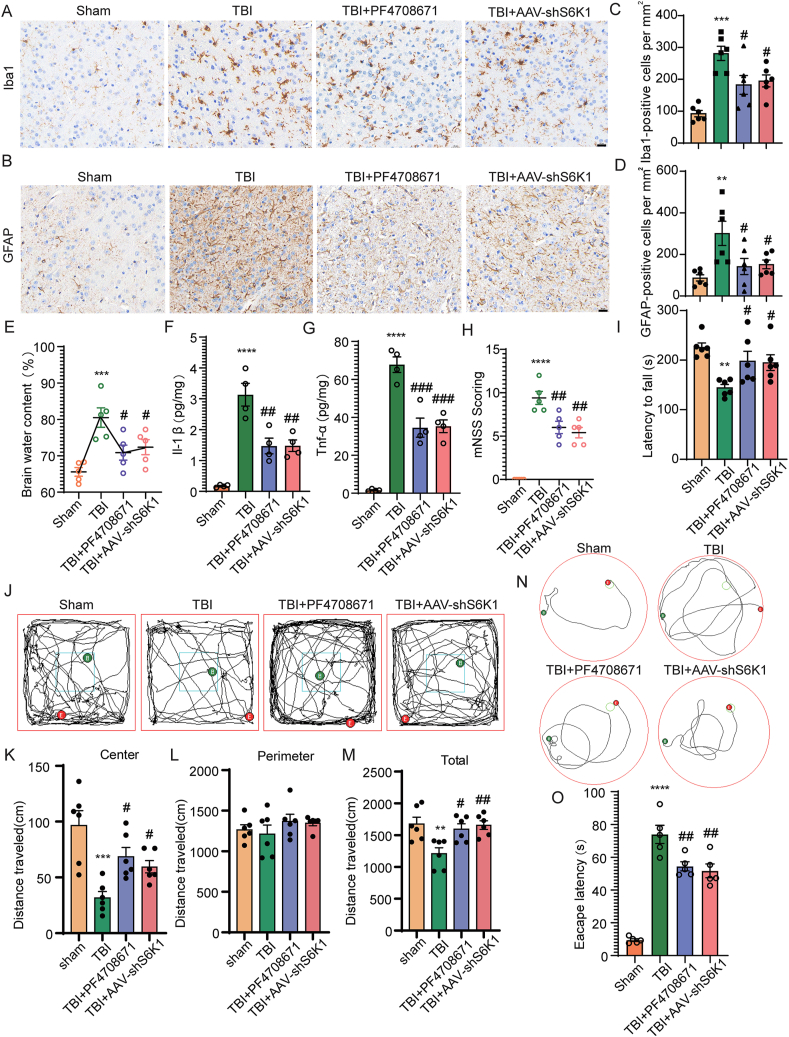
S6K1 inhibition alleviates neuro-inflammation and functional damage after TBI in mice. **(A, B)** Immunohistochemistry staining results showed increased activation of Iba1 and GFAP in the right cortex of mouse brains after TBI. This activation was reduced following treatment with an S6K1 inhibitor or *S6K1* knockdown, compared with the TBI group. **(C, D)** Statistical results of immunohistochemistry staining for the numbers of Iba1-positive microglia cells and GFAP-positive astrocytes. **(E)** TBI induced significant brain tissue edema, which was inhibited by treatment with S6K1 inhibitor and knockdown. **(F, G)** ELISA showed increased Tnf-α and Il-1β in mice after TBI, and decreased inflammation after treatment with S6K1 inhibitor and knockdown, compared with TBI. **(H)** mNSS showed impaired neurological performances in mice following TBI, and increased neurological functions after treatment with S6K1 inhibitor and knockdown, compared with TBI. **(I)** Rotarod test showed the decreased motor coordination of mice following TBI, and increased motor coordination after treatment with S6K1 inhibitor and knockdown, compared with TBI. **(J)** Open-field test results showed the increased anxiety and decreased motor function of mice following TBI, and decreased anxiety and increased motor function after treatment with S6K1 inhibitor and knockdown, compared with TBI. **(K**–**M)** Statistical results of open-field test showed the total travel distance and the distance traveled within the central square and perimeter. **(N)** Morris water maze test results showed the increased interval track and escape latency to find the platform during hidden platform trial of mice following TBI, and decreased escape latency and movement distance after treatment with S6K1 inhibitor and knockdown, compared with TBI. **(O)** Statistical results of Morris water maze tests showed the escape latency. *n* = 4, 5, or 6. *∗∗P* < 0.01, *∗∗∗P* < 0.001, and *∗∗∗∗P* < 0.0001 versus the Sham group. ^*#*^*P* < 0.05 and ^*##*^*P* < 0.01 versus the TBI group. Scale bar: 20 μm.

Given the reports that necroptosis is an inflammatory form of cell death, we evaluated alterations in inflammation following TBI injury in mice. After TBI, microglial cells and astrocytes were significantly activated, resulting in cell swelling and branching (Fig. 6A–D). The number of Iba1-positive microglial cells and GFAP-positive astrocytes increased after injury (Fig. 6A–D). After application of the S6K1 inhibitors PF4708671 and AAV-shS6K1, the number of activated microglial cells and astrocytes significantly decreased (Fig. 6A–D), suggesting that inhibition of S6K1 can alleviate neuroinflammation induced by TBI. In addition, TBI induced significant brain edema, which was alleviated by application of the S6K1 inhibitors PF4708671 and AAV-shS6K1 (Fig. 6E). ELISA further showed that inflammatory cytokines increased after TBI injury and decreased after application of the S6K1 inhibitors PF4708671 and AAV-shS6K1 (Fig. 6F and G). Together, these results suggest that S6K1 inhibition could alleviate neuroinflammation induced by TBI.

The functional changes were evaluated. TBI mice showed higher mNSS scores than sham-operated mice, which were lower in mice treated with the S6K1 inhibitors PF4708671 and AAV-shS6K1 (Fig. 6H). The rotarod test showed that the mice maintained a longer exercise time on the rod after S6K1 inhibition than the TBI group (Fig. 6I), indicating that the coordination and exercise ability of the mice improved after S6K1 inhibition. The open-field test results revealed that TBI mice showed a marked decrease in distance in the central area and total distance, which could be reversed by S6K1 inhibition (Fig. 6J–M), suggesting that TBI mice after S6K1 intervention have less anxiety and enhanced motor function. Finally, Morris water maze tests showed an increased distance traveled to reach the platform following TBI, which decreased after the application of the S6K1 inhibitors PF4708671 and AAV-shS6K1 (Fig. 6N and O). Together, these results suggest that S6K1 inhibition could alleviate functional damage induced by TBI.

Taken together, these results indicated that S6K1 inhibition alleviated neuroinflammation and functional damage in mice following TBI.

## Discussion

In this study, our findings showed a non-canonical regulated role of the “S6K1–SGK1” pathway in neuronal necroptosis following TBI. Our results first showed that the “S6K1–SGK1” pathway was activated during neuronal necroptosis. Then, inhibition of the “S6K1–SGK1” pathway could decrease necroptosis by regulating the MLKL activation. Finally, S6K1 inhibition alleviated neuronal necroptosis, neuroinflammation, and functional damage via SGK1 in mice after TBI.

Traditionally, mTORC1 is responsible for the initiation of protein synthesis, depending on its targets 4EBP1 and S6K1.^18^^,^^19^ Besides, under rich nutrients, mTORC1 also inhibits autophagy by phosphorylating its target ULK1.^18^^,^^19^ Recent studies have suggested that mTORC1 is involved in necroptosis.^7^^,^^20^^,^^21^ Other studies have indicated that ULK1 promotes RIP1 phosphorylation and facilitates autophagy, thereby inhibiting necroptosis.^22^^,^^23^ Along with these reports, our recent study discovered a positive regulatory loop between the “4EBP1–eIF4E” pathway and “RIP3–MLKL” that promotes neuronal necroptosis, which is consistent with other reports that RIP1-dependent necroptosis could enhance vasculogenic mimicry formation through increased expression of eIF4E in triple-negative breast cancer.^24^, ^25^, ^26^ However, the regulatory role of S6K1 in necroptosis remains unclear. Recent studies have indicated that RSK1/2, which belongs to the ribosomal AGC family, together with S6K1, can be activated in necroptotic cells through PDK1.^28^^,^^29^ Activated RSK1/2 is essential for stabilizing the necrosome complex during necroptosis.^28^^,^^29^ All these reports suggest that S6K1 may be an underlying regulator of necroptosis.

In this study, we showed that S6K1 inhibition decreases necroptosis by inhibiting MLKL activation and alleviating neuroinflammation and functional brain damage. Consistent with our results, it has been reported that the mTORC1–S6K1 pathway is phosphorylated and activated after ischemic stroke. The inhibition of the mTORC1–S6K1 pathway has potential protective effects against stroke injury.^38^^,^^39^ A similar finding was discovered for the cardioprotective actions of insulin-like growth factor 1 (IGF1), which could reduce cell death by inhibiting the mTORC1–S6K1 pathway.^40^ Further studies have shown that S6K1 inhibition protects cells against myocardial infarction via PDK1 phosphorylation.^41^ In addition, the role of S6K1 activation in promoting apoptosis has been discovered; elevated Rab1a can activate the mTORC1–S6K1 pathway, finally promoting cell apoptosis and aggravating osteoarthritis.^42^ The role of S6K1 in other types of programmed necrosis has been reported previously. High mobility group protein A2 (HMGA2) is highly expressed in pancreatic cancer tissues and correlates with inhibited ferroptosis.^43^^,^^44^ Studies have indicated that high HMGA2 levels inhibit the protein synthesis of glutathione peroxidase 4 (GPX4), a marker of ferroptosis, by reducing 4EBP1 and S6K1 activity, indicating that 4EBP1 and S6K signaling could modulate ferroptosis in cancer cell proliferation and metastasis.^43^^,^^45^^,^^46^ Taken together, these reports show that S6K1 may be a vital regulator of programmed cell death, including necroptosis, apoptosis, and ferroptosis, providing an important therapeutic method and direction for related diseases. However, whether S6K1 is a key regulator of cross-talk signaling in different types of programmed cell death requires further investigation.

SGK1 is a downstream effector of mTORC2 that promotes survival signaling in response to stress stimuli and plays an important role in various diseases, including cancer, hypertension, and neurodegenerative disorders.^32^^,^^33^ SGK1 activation has been suggested to restore damage during ischemic stroke.^47^ SGK1 inhibitors reduce neurotoxicity mediated by the activation of N-methyl-d-aspartate (NMDA) receptors and calcium overloading, ultimately inhibiting cell death and increasing infarct volume in stroke.^47^ The functions of mTORC2-related pathways in necroptosis have been revealed in recent years.^21^ mTORC2 is also involved in other types of programmed necrosis, such as pyroptosis and ferroptosis.^48^, ^49^, ^50^, ^51^ However, its role of SGK1 in necroptosis remains unclear. In this study, we found that SGK1 inhibition decreased necroptosis by inhibiting MLKL activation. Similar to our study, other reports have indicated that SGK1 expression also increases and peaks on day 3 after TBI. However, in that study, SGK1 regulated neuronal apoptosis through the GSK3β/β-catenin signaling pathway.^52^ Overall, our and other studies have shown that TBI induces the up-regulation of SGK1 expression, which indicates that it is a protective target in neuronal survival.

SGK1 dysfunction is related to other neurological diseases such as chronic stress, inhibition of glutamatergic excitability, and axonal damage in abnormal white matter.^53^^,^^54^ In addition, in other diseases such as unilateral ureteral obstruction, the expression of SGK1 is up-regulated in the contralateral kidney and is associated with the activation of the mineralocorticoid receptor.^55^ Blocking the mineralocorticoid receptor using spironolactone suppresses pyroptosis via SGK1.^56^ Other studies have reported that SGK1 overexpression has the opposite effect, inhibiting apoptosis through caspases upon ceramide stimuli.^57^ For example, SGK1 exerts neurotrophic effects on neuronal survival and axon regeneration.^58^ These studies indicate that SGK1 may play opposing roles in different contexts in which it is activated. Classical evidence indicates that the role of SGK1 in the C. *elegans* lifespan is conflicting.^59^ The SGK1 pathway inhibits stress-response transcription factors in the intestine to limit longevity. SGK1 functions in neurons and increases the lifespan at lower temperatures.^59^ Given the diverse effects of SGK1 on cells, it has been suggested that SGK1 is responsible for different contexts and should be further investigated.

**Figure 7:**
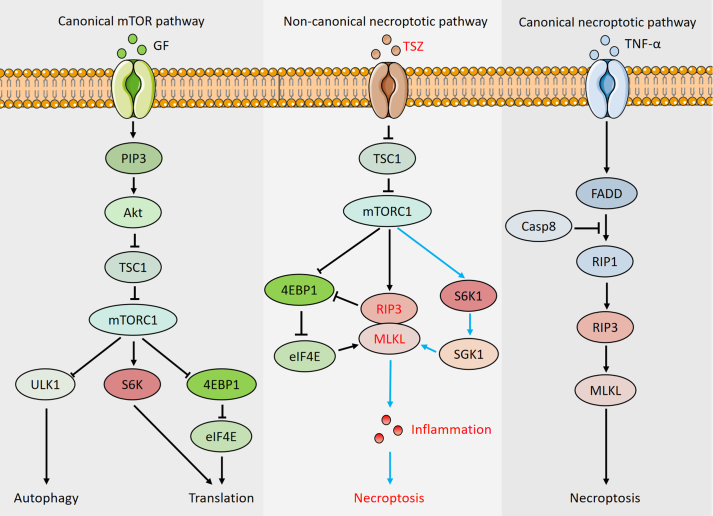
The schematic depicting the canonical and non-canonical mTORC1 and necroptotic pathway. **(A)** Canonical mTORC1 pathway. Traditionally, mTORC1 is known to regulate protein synthesis and autophagy by phosphorylating its three major substrates: 4EBP1, S6K, and ULK1. **(B)** Non-canonical role of “RSK1–SGK1” pathway in necroptosis, indicated by blue arrow. Under necroptotic stimuli, TSC1 inhibition could lead to RIP3 and MLKL activation through mTORC1, finally leading to necroptosis through “4EBP1–eIF4E" and “RSK1–SGK1” pathway. **(C)** Canonical RIP3/MLKL-mediated necroptosis. In traditional necroptotic pathway, caspase-8 could suppress FADD/RIP1/RIP3/MLKL-dependent necroptosis.

We further identified that S6K1 may regulate necroptosis by modulating SGK1 expression but not by binding to SGK1. Several studies have shown that PDK1 interacts with and activates S6K1, RSK, and SGK1 during insulin signaling,^60^^,^^61^ indicating an underlying interaction between S6K1 and SGK1. However, further experiments showed that both wild-type and phosphorylated S6K1 could bind to PDK1. PDK1 only interacted with phosphorylated SGK1 when the phosphorylation site of serine 422 was activated, but did not interact with wild-type SGK1.^33^, ^34^, ^35^ In the present study, we did not observe any changes in SGK1 phosphorylation during necroptosis. These results suggest that S6K1 does not bind to overexpressed wild-type SGK1 during necroptosis. Furthermore, it has been reported that S6K1 can regulate SAPK-interacting protein 1 (Sin1), which is a vital component of mTORC2 and is crucial for mTORC2 function, whereas mTORC2 cannot phosphorylate S6K.^62^^,^^63^ These reports are consistent with our findings that S6K1 is an up-regulator of SGK1 during necroptosis. Additionally, other reports have shown that S6K1 can phosphorylate PDK1 to inhibit insulin signaling,^31^^,^^64^ indicating that S6K1 is an upstream regulator of SGK1, a downstream molecule of PDK1. Taken together, our results indicate that S6K1 up-regulates SGK1 in the necroptotic process (Fig. 7), but not through its binding to SGK1.

TBI is a serious nervous system disorder with a high rate of death and disability; however, its pathophysiological mechanisms are poorly defined. In this study, we observed a novel function of the S6K1–SGK1 pathway in the regulation of necroptosis, which is unlike the classical roles of S6K1 and SGK1 in diverse cellular processes, such as protein synthesis, cell proliferation, apoptosis, and cellular stress response. Our current study strongly indicates that “S6K1–SGK1” may be a promising therapeutic target for necroptosis-mediated diseases. Furthermore, the clinical significance of this study needs to be clarified.

## Conclusion

In summary, our results demonstrate a non-canonical role of the “S6K1–SGK1” pathway in neuronal necroptosis following TBI in mice, which will provide a potential therapeutic target for necroptosis treatment in TBI and other necroptosis-related disorders.

## CRediT authorship contribution statement

**Shuchao Wang:** Writing – review & editing, Writing – original draft, Visualization, Validation, Supervision, Project administration, Methodology, Investigation, Funding acquisition, Formal analysis, Data curation, Conceptualization. **Yating Tan:** Writing – original draft, Project administration, Investigation, Formal analysis, Data curation. **Minghai Hu:** Investigation, Methodology, Project administration, Software, Writing – review & editing. **Meijuan Wang:** Writing – review & editing, Software, Methodology, Investigation, Data curation. **Lu Liang:** Project administration, Methodology, Investigation, Formal analysis, Data curation. **Xing Luo:** Software, Methodology, Investigation, Formal analysis, Data curation. **Dan Chen:** Writing – review & editing, Supervision, Project administration, Conceptualization. **Bing Jiang:** Writing – review & editing, Supervision, Project administration, Conceptualization. **Ceshi Chen:** Funding acquisition, Supervision, Writing – review & editing. **Jufang Huang:** Writing – review & editing, Supervision, Project administration, Funding acquisition, Conceptualization. **Kun Xiong:** Writing – review & editing, Project administration, Funding acquisition, Conceptualization.

## Data availability

All data in this study are available upon request by contacting the corresponding authors.

## Ethics declaration

All animal experiments were approved by the animal research committee of The Second Xiangya Hospital of Central South University (approval number: 2021136) in accordance with the U.S. National Institutes of Health (NIH) Guidelines for the Care and Use of Laboratory Animals.

## Funding

The study was supported by the 10.13039/501100001809National Natural Science Foundation of China (No. 82101126, 82572869, 82172196, 82372507, 81371011, 81671225), the 10.13039/501100004735Natural Science Foundation of Hunan Province, China (No. 2024JJ5472, 2021JJ40873), the Scientific Research Launch Project for new employees of the Second Xiangya Hospital of Central South University (China), and the Flexible Talent Introduction Program of Central South University (China).

## Conflict of interests

Ceshi Chen is the member of Genes & Diseases Editorial Board. To minimize bias, he/she was excluded from all editorial decision-making related to the acceptance of this article for publication. The remaining authors declared no conflict of interests.

## References

[1] Friberg S., Lindblad C., Zeiler F.A. (2024). Fluid biomarkers of chronic traumatic brain injury. Nat Rev Neurol.

[2] Turgeon A.F., Fergusson D.A., Clayton L. (2024). Liberal or restrictive transfusion strategy in patients with traumatic brain injury. N Engl J Med.

[3] Chen J., Shi Z., Chen Y., Xiong K., Wang Y., Zhang H. (2025). A CoQ10 analog ameliorates cognitive impairment and early brain injury after subarachnoid hemorrhage by regulating ferroptosis and neuroinflammation. Redox Biol.

[4] Glover H.L., Schreiner A., Dewson G., Tait S.W.G. (2024). Mitochondria and cell death. Nat Cell Biol.

[5] Newton K., Strasser A., Kayagaki N., Dixit V.M. (2024). Cell death. Cell.

[6] Hu X., Xu Y., Zhang H. (2022). Role of necroptosis in traumatic brain and spinal cord injuries. J Adv Res.

[7] Xu Y., Geng Y., Wang H. (2023). Cyclic helix B peptide alleviates proinflammatory cell death and improves functional recovery after traumatic spinal cord injury. Redox Biol.

[8] Huang K., Zhang Q., Wan H. (2025). TAK1 at the crossroads of multiple regulated cell death pathways: from molecular mechanisms to human diseases. FEBS J.

[9] Wan H., Yang Y.D., Zhang Q. (2024). VDAC1, as a downstream molecule of MLKL, participates in OGD/R-induced necroptosis by inducing mitochondrial damage. Heliyon.

[10] Wan H, Ban X, He Y, et al. Voltage-dependent anion channel 1 oligomerization regulates PANoptosis in retinal ischemia-reperfusion injury. Neural Regen Res. 2026;21(4):1652-1664. 10.4103/NRR.NRR-D-24-00674 PMC12407563 39819824

[11] Zhao P., Wei Y., Sun G. (2022). Fetuin-A alleviates neuroinflammation against traumatic brain injury-induced microglial necroptosis by regulating Nrf-2/HO-1 pathway. J Neuroinflammation.

[12] Wehn A.C., Khalin I., Duering M. (2021). RIPK1 or RIPK3 deletion prevents progressive neuronal cell death and improves memory function after traumatic brain injury. Acta Neuropathol Commun.

[13] Plemel J.R., Caprariello A.V., Keough M.B. (2017). Unique spectral signatures of the nucleic acid dye acridine orange can distinguish cell death by apoptosis and necroptosis. J Cell Biol.

[14] Zhang H., Ni W., Yu G. (2023). 3, 4-Dimethoxychalcone, a caloric restriction mimetic, enhances TFEB-mediated autophagy and alleviates pyroptosis and necroptosis after spinal cord injury. Theranostics.

[15] Hu X., Zhang H., Zhang Q., Yao X., Ni W., Zhou K. (2022). Emerging role of STING signalling in CNS injury: inflammation, autophagy, necroptosis, ferroptosis and pyroptosis. J Neuroinflammation.

[16] You Z., Savitz S.I., Yang J. (2008). Necrostatin-1 reduces histopathology and improves functional outcome after controlled cortical impact in mice. J Cereb Blood Flow Metab.

[17] Chen T., Zhu J., Wang Y.H., Hang C.H. (2020). Arc silence aggravates traumatic neuronal injury via mGluR1-mediated ER stress and necroptosis. Cell Death Dis.

[18] Battaglioni S., Benjamin D., Wälchli M., Maier T., Hall M.N. (2022). mTOR substrate phosphorylation in growth control. Cell.

[19] Gros F., Muller S. (2023). The role of lysosomes in metabolic and autoimmune diseases. Nat Rev Nephrol.

[20] Zhang J., Liu D., Fu P. (2022). Social isolation reinforces aging-related behavioral inflexibility by promoting neuronal necroptosis in basolateral amygdala. Mol Psychiatry.

[21] Xie Y., Zhao Y., Shi L. (2020). Gut epithelial TSC1/mTOR controls RIPK3-dependent necroptosis in intestinal inflammation and cancer. J Clin Invest.

[22] Tang S., Liang C., Hou W. (2023). ATP7B R778L mutant hepatocytes resist copper toxicity by activating autophagy and inhibiting necroptosis. Cell Death Discov.

[23] Wu W., Wang X., Sun Y. (2021). TNF-induced necroptosis initiates early autophagy events via RIPK3-dependent AMPK activation, but inhibits late autophagy. Autophagy.

[24] Li F., Sun H., Yu Y. (2023). RIPK1-dependent necroptosis promotes vasculogenic mimicry formation via eIF4E in triple-negative breast cancer. Cell Death Dis.

[25] Wang S., Xu M. (2023). RIP3/MLKL regulates necroptosis via activating 4EBP1-eIF4E pathway. Zhong Nan Da Xue Xue Bao Yi Xue Ban.

[26] Wang S., Zhang Y., Wang M. (2025). Noncanonical feedback loop between “RIP3-MLKL” and “4EBP1-eIF4E” promotes neuronal necroptosis. MedComm (2020).

[27] Wang M., Wan H., Wang S. (2020). RSK3 mediates necroptosis by regulating phosphorylation of RIP3 in rat retinal ganglion cells. J Anat.

[28] Yang Z.H., Wu X.N., He P. (2020). A non-canonical PDK1-RSK signal diminishes pro-caspase-8-mediated necroptosis blockade. Mol Cell.

[29] He P., Ai T., Qiao M., Yang Z.H., Han J. (2024). Phosphorylation of caspase-8 by RSKs via organ-constrained effects controls the sensitivity to TNF-induced death. Cell Death Discov.

[30] Frödin M., Antal T.L., Dümmler B.A. (2002). A phosphoserine/threonine-binding pocket in AGC kinases and PDK1 mediates activation by hydrophobic motif phosphorylation. EMBO J.

[31] Jiang Q., Zhang X., Dai X. (2022). S6K1-mediated phosphorylation of PDK1 impairs AKT kinase activity and oncogenic functions. Nat Commun.

[32] Lizcano J.M., Deak M., Morrice N. (2002). Molecular basis for the substrate specificity of NIMA-related kinase-6 (NEK6). Evidence that NEK6 does not phosphorylate the hydrophobic motif of ribosomal S6 protein kinase and serum- and glucocorticoid-induced protein kinase *in vivo*. J Biol Chem.

[33] Biondi R.M., Kieloch A., Currie R.A., Deak M., Alessi D.R. (2001). The PIF-binding pocket in PDK1 is essential for activation of S6K and SGK, but not PKB. EMBO J.

[34] Tremblay F., Brûlé S., Um S.H. (2007). Identification of IRS-1 *Ser*-1101 as a target of S6K1 in nutrient- and obesity-induced insulin resistance. Proc Natl Acad Sci U S A.

[35] Um S.H., Frigerio F., Watanabe M. (2004). Absence of S6K1 protects against age- and diet-induced obesity while enhancing insulin sensitivity. Nature.

[36] Chen Y., Long T., Chen J. (2024). WTAP participates in neuronal damage by protein translation of NLRP3 in an m6A-YTHDF1-dependent manner after traumatic brain injury. Int J Surg.

[37] Chi O.Z., Mellender S.J., Kiss G.K. (2020). Lysophosphatidic acid increased infarct size in the early stage of cerebral ischemia-reperfusion with increased BBB permeability. J Stroke Cerebrovasc Dis.

[38] Jiang R.H., Xu X.Q., Wu C.J. (2018). The CD40/CD40L system regulates rat cerebral microvasculature after focal ischemia/reperfusion via the mTOR/S6K signaling pathway. Neurol Res.

[39] Xie R., Wang P., Ji X., Zhao H. (2013). Ischemic post-conditioning facilitates brain recovery after stroke by promoting Akt/mTOR activity in nude rats. J Neurochem.

[40] Troncoso R., Vicencio J.M., Parra V. (2012). Energy-preserving effects of IGF-1 antagonize starvation-induced cardiac autophagy. Cardiovasc Res.

[41] Di R., Wu X., Chang Z. (2012). S6K inhibition renders cardiac protection against myocardial infarction through PDK1 phosphorylation of Akt. Biochem J.

[42] Chen Z., Tang M., Wu Z. (2025). Increased Rab1a accelerates osteoarthritis by inhibiting autophagy via activation of the mTORC1-S6K pathway. J Adv Res.

[43] Luo Z., Zheng Q., Ye S. (2024). HMGA2 alleviates ferroptosis by promoting GPX4 expression in pancreatic cancer cells. Cell Death Dis.

[44] Chen J., Wang Y., Li M. (2024). Netrin-1 alleviates early brain injury by regulating ferroptosis via the PPARγ/Nrf2/GPX4 signaling pathway following subarachnoid hemorrhage. Transl Stroke Res.

[45] Dike P.E., Hwang B.J., Campbell T., Awolowo M., Elliott B., Odero-Marah V. (2024). HMGA2 regulates GPX4 expression and ferroptosis in prostate cancer cells. Biochem Biophys Res Commun.

[46] Campbell T., Hawsawi O., Henderson V. (2023). Novel roles for HMGA2 isoforms in regulating oxidative stress and sensitizing to RSL3-Induced ferroptosis in prostate cancer cells. Heliyon.

[47] Inoue K., Leng T., Yang T., Zeng Z., Ueki T., Xiong Z.G. (2016). Role of serum- and glucocorticoid-inducible kinases in stroke. J Neurochem.

[48] Xiang C., Chen L., Zhu S. (2024). CRLF1 bridges AKT and mTORC2 through SIN1 to inhibit pyroptosis and enhance chemo-resistance in ovarian cancer. Cell Death Dis.

[49] Wang Y., Tian Q., Hao Y. (2022). The kinase complex mTORC2 promotes the longevity of virus-specific memory CD4^+^ T cells by preventing ferroptosis. Nat Immunol.

[50] Wang G., Chen L., Qin S. (2022). Cystine induced-mTORC2 activation through promoting Sin1 phosphorylation to suppress cancer cell ferroptosis. Mol Nutr Food Res.

[51] Chen J., Shi Z., Zhang C., Xiong K., Zhao W., Wang Y. (2024). Oroxin A alleviates early brain injury after subarachnoid hemorrhage by regulating ferroptosis and neuroinflammation. J Neuroinflammation.

[52] Wu X., Mao H., Liu J. (2013). Dynamic change of SGK expression and its role in neuron apoptosis after traumatic brain injury. Int J Clin Exp Pathol.

[53] Miyata S., Taniguchi M., Koyama Y. (2016). Association between chronic stress-induced structural abnormalities in Ranvier nodes and reduced oligodendrocyte activity in major depression. Sci Rep.

[54] Taub D.G., Awal M.R., Gabel C.V. (2018). O-GlcNAc signaling orchestrates the regenerative response to neuronal injury in *Caenorhabditis elegans*. Cell Rep.

[55] Wang C.H., Wang Z., Liang L.J. (2017). The inhibitory effect of eplerenone on cell proliferation in the contralateral kidneys of rats with unilateral ureteral obstruction. Nephron.

[56] Ma X., Qiang P., Chen G., Wang Z., Wang X., Xu Q. (2021). Huoxue Jiedu Huayu formula alleviates cell pyroptosis in contralateral kidneys of 6-month-old UUO rats through the NLRP3/caspase-1/IL-1β pathway. Evid Based Complement Alternat Med.

[57] Pastore D., Della-Morte D., Coppola A. (2015). SGK-1 protects kidney cells against apoptosis induced by ceramide and TNF-α. Cell Death Dis.

[58] Chen X., Tagliaferro P., Kareva T., Yarygina O., Kholodilov N., Burke R.E. (2012). Neurotrophic effects of serum- and glucocorticoid-inducible kinase on adult murine mesencephalic dopamine neurons. J Neurosci.

[59] Mizunuma M., Neumann-Haefelin E., Moroz N., Li Y., Blackwell T.K. (2014). mTORC2-SGK-1 acts in two environmentally responsive pathways with opposing effects on longevity. Aging Cell.

[60] Chun J., Kwon T., Lee E., Suh P.G., Choi E.J., Kang S.S. (2002). The Na^+^/H^+^ exchanger regulatory factor 2 mediates phosphorylation of serum- and glucocorticoid-induced protein kinase 1 by 3-phosphoinositide-dependent protein kinase 1. Biochem Biophys Res Commun.

[61] Leroux A.E., Schulze J.O., Biondi R.M. (2018). AGC kinases, mechanisms of regulation and innovative drug development. Semin Cancer Biol.

[62] Zhang J., Gao Z., Yin J., Quon M.J., Ye J. (2008). S6K directly phosphorylates IRS-1 on *Ser*-270 to promote insulin resistance in response to TNF-(alpha) signaling through IKK2. J Biol Chem.

[63] Jhanwar-Uniyal M., Amin A.G., Cooper J.B., Das K., Schmidt M.H., Murali R. (2017). Discrete signaling mechanisms of mTORC1 and mTORC2: connected yet apart in cellular and molecular aspects. Adv Biol Regul.

[64] Leroux A.E., Biondi R.M. (2023). The choreography of protein kinase PDK1 and its diverse substrate dance partners. Biochem J.

